# Ciprofloxacin-Releasing ROS-Sensitive Nanoparticles Composed of Poly(Ethylene Glycol)/Poly(D,L-lactide-co-glycolide) for Antibacterial Treatment

**DOI:** 10.3390/ma14154125

**Published:** 2021-07-24

**Authors:** Jaeik Song, Min-Suk Kook, Byung-Hoon Kim, Young-IL Jeong, Kyung-Jin Oh

**Affiliations:** 1 Department of Urology, Chonnam National University Medical School, Chonnam National University Hospital, Gwangju 61469, Korea; puzzlelist@naver.com; 2 Department of Maxillofacial Surgery, School of Dentistry, Chonnam National University, Gwangju 61186, Korea; omskook@chonnam.ac.kr; 3 Department of Dental Materials, School of Dentistry, Chosun University, Gwangju 61452, Korea; kim5055@chosun.ac.kr (B.-H.K.); nanomed@naver.com (Y.-I.J.)

**Keywords:** urethritis, infectious disease, reactive oxygen species, stimuli-sensitive nanoparticles, redox-responsive

## Abstract

Since urinary tract infections (UTIs) are closely associated with oxidative stress, we developed ROS-sensitive nanoparticles for ciprofloxacin (CIP) delivery for inhibition of UTI. Poly(D,L-lactide-co-glycolide) (PLGA)- selenocystamine (PLGA-selenocystamine) conjugates were attached to methoxypoly(ethylene glycol) (PEG) tetraacid (TA) (TA-PEG) conjugates to produce a copolymer (abbreviated as LGseseTAPEG). Selenocystamine linkages were introduced between PLGA and TA to endow reactive oxygen species (ROS) sensitivity to nanoparticles. CIP-incorporated nanoparticles of LGseseTAPEG copolymer were fabricated by W/O/W/W emulsion method. CIP-incorporated nanoparticles responded to H_2_O_2_ and then their morphologies were disintegrated by incubation with H_2_O_2_. Furthermore, particle size distribution of nanoparticles was changed from mono-modal distribution pattern to multi-modal distribution pattern by addition of H_2_O_2_. CIP release from nanoparticles of LGseseTAPEG copolymer was faster in the presence of H_2_O_2_ than in the absence of it. In antibacterial study using *Escherichia coli* (*E. coli*), free CIP and free CIP plus empty nanoparticles showed dose-dependent inhibitory effect against growth of bacteria while CIP-incorporated nanoparticles have less antibacterial activity compared to free CIP. These results were due to that CIP-incorporated nanoparticles have sustained release properties. When free CIP or CIP-incorporated nanoparticles were introduced into dialysis membrane to mimic in vivo situation, CIP-incorporated nanoparticles showed superior antibacterial activity compared to free CIP. At cell viability assay, nanoparticles of LGseseTAPEG copolymer have no acute cytotoxicity against L929 mouse fibroblast cells and CCD986sk human skin fibroblast cells. We suggest LGseseTAPEG nanoparticles are a promising candidate for CIP delivery.

## 1. Introduction

A urinary tract infection (UTI), which is a bacterial infection in the part of urinary system, is one of most common infectious disease in human healthcare problems [1,2]. Although a UTI can occur anywhere in the urinary tract system such as kidneys, ureters, bladder and urethra, it most frequently develops in the urethra and bladder. Especially, UTI is also frequently associated with urinary catheters, most common devices for urinary tract treatment, and urinary catheter-associated UTIs may causes urinary dysfunction [3,4]. These issues cause significant problem in human health care morbidity [3,4,5]. Furthermore, oxidative stress can be elevated due to the UTIs, i.e., lipid peroxidation levels were increased in urine samples while catalase and superoxide dismutase activities were decreased [6]. Then, oxidative stress by UTIs causes oxidative damage in the urinary system resulting in urinary dysfunction [7,8]. To solve these problems, diverse types of antimicrobial agents including fluoroquinolone antibiotics have been used to treat UTIs [4,5]. However, the increase of pathogen drug resistance problems is also problematic in the clinic [9]. Among various antibiotics, the antibacterial efficacy of ciprofloxacin (CIP) is well-established in the treatment of UTI and various other infectious diseases [10,11,12]. CIP for antibacterial treatment is currently available as twice daily oral administration form and a once-daily extended release formulation [13]. However, these may disturb the normal intestinal environment, i.e., oral administration of CIP affects to the normal intestinal microflora, resulting in diarrhea and opportunistic infections [14]. Therefore, novel drug delivery systems are required to treat UTIs effectively.

Nanoparticle-based drug delivery systems have been extensively investigated to enhance the therapeutic efficacy of bioactive agents, anticancer agents, antibiotics, etc. [15,16,17]. Especially, stimuli-sensitive nanoparticles have been spotlighted for the last two decades since stimuli-sensitive drug delivery to specific sites of the body enables conventional drugs to target diseased cells or tissues with some response to a stimulus [18,19,20,21]. For example, nanoparticles composed of hyaluronic acid and poly(L-histidine) copolymer having disulfide linkages respond to the acidic pH/redox potential of tumors and then deliver anticancer drugs against tumors in a site-specific manner [19]. Stimuli-sensitive nanoparticles efficiently deliver CIP in an on-demand manner in infectious microenvironment models in vivo and thus show improved therapeutic efficacy compared to free CIP [21,22,23]. Alomary and Ansary reported that proanthocyanin-capped biogenic TiO_2_ nanoparticles showed inhibitory behavior against biofilm formation through ROS generation [24]. Among the various stimuli, oxidative stress in an infectious microenvironment or an inflammation environment can be feasible targets for therapeutics since the reactive oxygen species (ROS) level is known to be elevated in the region of bacterial infection or site of inflammation [6,25,26]. Kurutas et al., reported that lipid peroxidation levels were elevated in the urine samples with decreased levels of catalase and superoxide dismutase [6]. Furthermore, UTIs are known to aggravate oxidative stress in diabetic patients [26]. These issues may provide possibilities for oxidative species-mediated drug delivery systems against infectious disease such as UTIs.

In this study, we fabricated CIP-encapsulated nanoparticles using a star-shaped copolymer composed of methoxy poly(ethylene glycol) (mPEG) and poly(D,L-lacide-co-glycolide) (PLGA) having a diselenide linkage and a tetraacid (abbreviated as LGseseTAPEG). Since diselenide linkages can be cleaved under oxidative stress, these nanoparticles can be used to deliver CIP in a ROS-specific manner against UTI [27]. We studied ROS-sensitivity and ROS-sensitive drug release of LGseseTAPEG nanoparticles, and their antibacterial activity.

## 2. Materials and Methods

### 2.1. Materials

Poly(D,L-lactide-co-glycolide) (Resomer^®^ RG 502H, Evonik Ind. AG, Rellinghauser Straße 1—11, 45128 Essen, Germany, M.W.: 8000 g/mol) having a free carboxylic acid group at one end of the polymer was supplied by Evonik Ind. MePEG NH_2_ (Molecular weight (M.W.): 5000 g/mol) was supplied by SunBio Co. Ltd. (Seoul, Korea). TA was purchased from Frontier Scientific Co. Ltd. (Logan, UT, USA). Ciprofloxacin HCl (CIP), N-(3-dimethylaminopropyl)-N′-ethylcarbodiimide hydrochloride (EDAC), N-hydroxysuccinimide (NHS), dimethylsulfoxide-d_6_ (DMSO-d_6_) form, deuterium oxide (D_2_O), selenocystamine dihydrochloride, 3-[4,5-dimethylthiazol-2-yl]-2,5-diphenyltetrazolium bromide (MTT), triethylamine (TEA, 99%, density = 0.726 g/mL at 25 °C) and Luria-Bertani (LB) broth were purchased from Sigma-Aldrich Co. (St. Louis, MO, USA). Dialysis membrane (molecular weight cut-off: 2000 g/mol, 8000 g/mol) was purchased from Spectrum Lab., Inc. (New Brunswick, NJ, USA). All organic solvents used such as DMSO, ethyl alcohol, methylene chloride and chloroform were HPLC grade.

### 2.2. Synthesis of LGseseTAPEG Copolymer

#### 2.2.1. TA-PEG Conjugates

TA (46.7 mg, 0.11 mM) was dissolved in 10 mL DMSO with equal amount of EDAC (21 mg, 0.11 mM) and NHS (12.7 mg, 0.11 mM). This was stirred magnetically for 3 h and then mPEG-NH_2_ (500 mg, 0.1 mM) was added to this reaction mixture, which was magnetically stirred for 24 h. Following this, the reaction mixture was placed in a dialysis membrane (MWCO, 2000 g/mol) and dialyzed against deionized water for 2 days. To prevent saturation, the water was exchanged every 3 h and, after that, the dialyzed solution was lyophilized for more than 2 days. The reaction yield was higher than 95.4% (*w*/*w*) by measurement of the mass of each chemical. The yield was calculated using the following expression:

Yield (%, *w*/*w*) = [Weight of final product/(weight of TA + weight of PEG)] × 100. (1)

#### 2.2.2. PLGA-Selenocystamine Conjugates (LGsese)

PLGA (2400 mg, 0.3 mM) was dissolved in 20 mL DMSO with EDAC (57.5 mg, 0.3 mM) and NHS (34.5 mg, 0.3 mM) and then stirred magnetically for 3 h. To this reaction mixture an excess of selenocystamine HCl (479 mg 1.5 mM) was added along with a trace amount of TEA (3 mM, 0.42 mL). The mixture was stirred magnetically for 24 h and then the resulting solution was placed in a dialysis membrane (MWCO: 2000 g/mol) to remove unreacted chemicals by dialysis against deionized water for 2 days with exchange of water every 3 h. The resulting solution was finally lyophilized for 3 days.

#### 2.2.3. LGseseTAPEG Copolymer

TA-PEG conjugate (545 mg) dissolved in 20 mL DMSO was mixed with EDAC (57.5 mg, 0.3 mM) and NHS (34.5 mg, 0.3 mM). Then, this mixture was magnetically stirred for 3 h and, following this, 2400 mg of LGsese conjugates was added. This reaction mixture was further stirred for 1 day and then dialyzed against deionized water for 2 days using a dialysis membrane (MWCO: 8000 g/mol) to remove unreacted products. The resulting solution was lyophilized for more than 3 days. To remove unreacted chemicals and byproducts, lyophilized solid was washed with ethyl alcohol and then filtered through filter paper (Whatman No. 6). The product was dried under vacuum for 2 days. The yield was calculated using the following equation:

Yield (%, *w*/*w*) = [(weight of TA PEG conjugates + weight of LGsese conjugates)/weight of LGseseTAPEG conjugates] × 100. (2)

The final yield of LGseseTAPEG copolymers was higher than 94% by weight.

### 2.3. Analysis of LGseseTAPEG Copolymer

Synthesis of conjugates and copolymer was confirmed by ^1^H nuclear magnetic resonance (NMR) spectroscopy (500 MHz superconducting Fourier transform-NMR spectrometer, Varian Unity Inova; Varian Inc., Santa Clara, CA, USA). For ^1^H-NMR analysis, PLGA, PEG, TA-PEG conjugates, PLGA-selenocystamine conjugates and LGseseTAPEG copolymer were dissolved in DMSO-d_6_ form. Selenocystamine was dissolved in D_2_O and mixed with DMSO-d_6_ form (D_2_O/DMSO-d_6_ form = 1/1, *v*/*v*).

The morphology of LGseseTAPEG nanoparticles was observed with a transmission electron microscope (TEM, H-7600, Hitachi Instruments Ltd., Tokyo, Japan). Aqueous nanoparticle solution was placed onto carbon film coated copper grid and then this was dried in room temperature. Nanoparticles were observed at 80 kV.

Particle size of nanoparticles (nanoparticle solution in phosphate-buffered saline (PBS, 0.01 M, pH 7.4): 1 mg/mL) was measured with a Nano-ZS system (Malvern, Worcestershire, UK). Particle sizes were measured three times independently and expressed as mean ± standard deviation (S.D.).

### 2.4. Preparation of CIP-Incorporated LGseseTAPEG Nanoparticles

CIP-incorporated nanoparticles were fabricated as follows: LGseseTAPEG copolymer (100 mg) was dissolved in 10 mL of DCM and CIP (10 mg or 20 mg) was dissolved in 1 mL deionized water. These solutions were mixed and vigorously sonicated with ultrasonicator (40 W, 1 min, Vibracell VCX 400, Sonics & Materials Inc., Newtown, CT, USA) to make a water in oil (W/O) emulsion. This solution was poured into 15 mL of aqueous PVA solution (1%, *w*/*v*) and then vigorously homogenized (HG-15A, Daihan Scientific, Seoul, Korea) at 15,000 rpm for 1 min. This was sonicated again with ultrasonicator to make water-in-oil-in water (W/O/W). This emulsion solution was poured into 50 mL PVA solution (0.5%, *w*/*v*) and then stirred with top-loading stirrer at 1000 rpm (Direct Driven Digital Stirrer, SS-11D, Young HANA Tech. Co., Seoul, Korea) for 90 min. Following this, this solution was centrifuged to harvest CIP-incorporated nanoparticles using vacuum high speed centrifuge at 15,000 rpm (Supra 30K, Hanil Science Industrial Co. Ltd., Seoul, Korea). To remove surfactant or unincorporated drugs, harvested nanoparticles were distributed in deionized water once more and then harvested again using a vacuum high-speed centrifuge at 15,000 rpm. These were lyophilized more than 2 days.

To measure CIP contents in the nanoparticles, 10 mg lyophilized solids were dissolved in 5 mL DCM and, after that, 2 mL of deionized water was added. This was magnetically stirred for more than 5 h and then 0.5 mL of water phase was used to measure CIP concentration at 277 nm using UV-VIS spectrophotometer (UV-VIS spectrophotometer 1601, Shimadzu Co. Tokyo, Japan) at 340 nm. Drug contents were calculated as follows:

Drug contents = (drug weight/nanoparticle weight) × 100. Loading efficiency = (Initial feeding amount of drug weight/remained drug weight in the nanoparticles) × 100.(3)

### 2.5. Drug Release from Nanoparticles

CIP release from nanoparticles was measured as follows: nanoparticles (10 mg) were distributed in 3 mL of phosphate buffered saline (PBS, pH 7.4, 0.01 M) and then put into a dialysis membrane (MWCO, 8000 g/mol). Following this, this was introduced into 50 mL Falcon tube with 47 mL of PBS and then stirred at 100 rpm (37 °C). Whole media were exchanged at predetermined time intervals to prevent saturation of drug and then released drug was measured with a UV-spectrophotometer (UV-1601, Shimadzu Co. Ltd.) at 277 nm. The following expression was used for the calculations:

Total released = [(Cumulative amount of released drug/total drug weight in the nanoparticles) × 100].(4)

### 2.6. Antibacterial Activity of CIP-Incorporated Nanoparticles In Vitro

Stock solutions were prepared and dilutions were made according to the Clinical Laboratory Standards Institute-CLSI (formerly NCCLS) M7-A6 method (15 National Committee for Clinical Laboratory Standards 2003). *Escherichia coli* (*E. coli*) were provided by Korea Collection for Type Cultures (KCTC, Jeongeup-si, Korea). Following subcultures from frozen stock, antimicrobial agents or nanoparticles were added to solutions of microorganisms at various concentrations. All experiments were performed in triplicate on separate days. Bacterial growth was determined by reading optical density at 600 nm (UV-spectrophotometer 1201, Shimadzu Co. Ltd.) after 1 day.

To mimic in vivo bacterial growth under sustained drug release from nanoparticles, CIP in PBS (0.1 mg CIP/0.5 mL PBS) was introduced into dialysis membrane (MWCO: 8000 g/mol). Nanoparticles in PBS solution (0.1 mg as a CIP concentration in 0.5 mL PBS) was also introduced into dialysis membrane. Same quantity of empty nanoparticles or empty nanoparticles with free CIP were also introduced into dialysis membrane to compare. For control treatment, 0.5 mL PBS in dialysis membrane was used. These were put into 10 mL of *E. coli* (1 × 10^6^/mL, LB broth). To mimic the in vivo state of drug or nanoparticles, half (5 mL) of culture media was exchanged with fresh media at every 30 min intervals for 3 h, 1 h intervals for 6 h and then at 2 h intervals for 15 h. After that, bacterial growth was determined by reading the optical density at 600 nm.

For treatment of free CIP, CIP-incorporated nanoparticles and free CIP + empty nanoparticles CIP was adjusted to similar concentration from the calculation of drug contents. Furthermore, empty nanoparticles for treatment of free CIP + empty nanoparticles were calculated from the result of drug content and adjusted empty nanoparticles similar to real polymer weight in the CIP-incorporated nanoparticles.

### 2.7. Cell Cytotoxcity of LGseseTAPEG Copolymer Nanoparticles In Vitro

L929 mouse fibroblast cells and CCD986sk human skin fibroblast cells obtained from Korean Cell Line Bank (KCLB, Korean Cell Line Bank Co. Ltd., Seoul, Korea) were used to test the intrinsic toxicity of LGseseTAPEG copolymer nanoparticles. L929 cells were cultured under 5% CO_2_ incubator at 37 °C. L929 cells were maintained and sub-cultured with RPMI1640 (Gibco^®^, Grand Island, NY, USA) supplemented with 10% heat-inactivated fetal bovine serum (FBS) (Gibco^®^, Life Technologies Co.) and 1% penicillin/streptomycin. CCD986sk cells were maintained in IMDM supplemented with 10% FBS and 1% penicillin/streptomycin.

For cell viability assay, 2 × 10^4^ cells were seeded in 96 well plates and then cultured overnight in 5% CO_2_ at 37 °C. Nanoparticles composed of LGseseTAPEG copolymer without CIP (empty nanoparticles) were fabricated as described above. Nanoparticles were sterilized with a 1.2 μm syringe filter (Sterile Millex^®^, Merck KGaA, Darmstadt, Germany) and then diluted with serum-free media. Nanoparticles were applied to cells in 96 well plates and then incubated in a 5% CO_2_ incubator for 24 h. After that, media were removed from the 96 well plates and then replaced with serum-free media containing MTT reagent (0.5 mg/mL) followed by incubation for 4 h at 37 °C. After that, supernatants were removed and 100 μL of DMSO was added to each wells to dissolved viable cells. The cell viability was estimated by measurement of the absorbance at 570 nm (Infinite M200 pro microplate reader, Tecan, Mannedorf, Switzerland).

### 2.8. Statistical Analysis

Statistical analysis of the results was evaluated with the Student *t*-test and the *p* values lower than 0.01 were considered as a significant value of statistical analysis.

## 3. Results and Discussion

Urinary tract infections are known to induce oxidative stress and then this state aggravates the disease symptoms [6,26]. *E. coli,* a common uropathogen, responds to an oxidative stress environment according to physiological changes and the level of the anti-oxidant molecule L-glutathione (GSH) is decreased under the stress conditions [28]. This feature of UTIs can provide a targeting motive for antibiotic-incorporated nanoparticles. Quinolone antibiotics such as CIP, which is a typical antibiotic used for UTI, may suppress human fibroblast cells [29].

### 3.1. Synthesis of LGseseTAPEG Copolymer

Based on the fact that oxidative stress is increased in UTIs, we have designed a ROS-sensitive nanoparticulate delivery system of CIP for the treatment of UTI. Since diselenide linkages respond to ROS and can be broken in an oxidative stress environment, diselenide linkages were introduced in the copolymer and ROS-sensitive nanoparticles fabricated [30,31].

**Figure 1:**
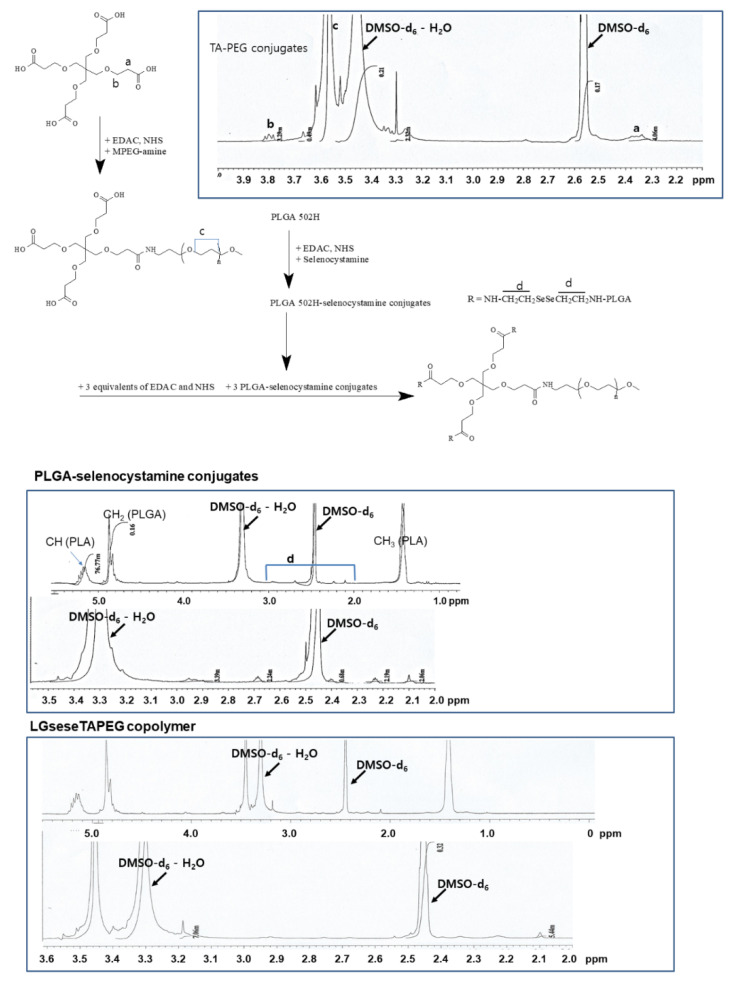
Synthesis scheme and ^1^H-NMR spectra of LGseseTAPEG copolymer.

PEG was attached to the carboxylic group via carbodiimide chemistry (TA-PEG conjugates) as shown in Figure 1. Unreacted TA was removed by a dialysis procedure and TA-PEG conjugates was used for analysis or as copolymer conjugates. As shown in Figure 1, the ethylene protons of PEG were observed in the ^1^H-NMR spectrum at about 3.6 ppm while specific peaks of TA were observed at 2.3–2.4 ppm and 3.7–3.9 ppm. Based on a comparison of peaks between the ethylene proton of PEG and the 3.8 ppm peak, the substitution yield of TA in the TA-PEG conjugates was estimated as 0.92 TA/1 PEG.

To synthesize PLGA-selenocystamine conjugates (LGsese), excess selenocystamine was reacted with PLGA and unreacted chemicals or byproducts were then removed by dialysis as reported by other researchers [32,33,34]. Specific peaks of PLGA were observed at 1.4~1.5 ppm and 4.6~5.4 ppm. Specific peaks of selenocystamine were observed at 1.8~3.0 ppm. PLGA-selenocystamine conjugates were conjugated again with TA-PEG conjugates to produce LGseseTAPEG copolymer as shown in Figure 1. Specific peaks of each component such as PEG, TA, PLGA and selenocystamine was observed between 1.4~5.4 ppm, indicating that LGseseTAPEG copolymer was successfully synthesized.

**Figure 2:**
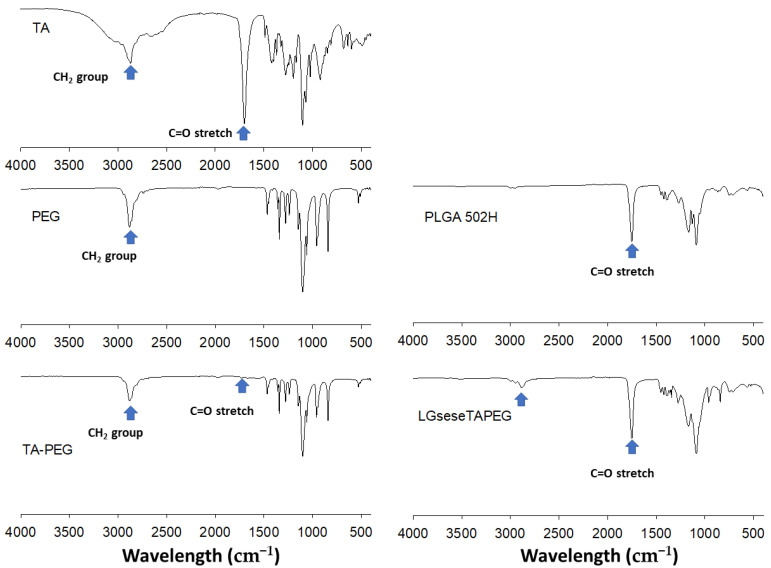
FT-IR spectra of LGseseTAPEG copolymer.

Figure 2 shows FT-IR spectra of LGseseTAPEG and each component. As shown in Figure 2, C=O and CH_2_ stretching bands of TA were observed at about 1720 cm^−1^ and 2800 cm^−1^, respectively. These peaks were also observed in TA-PEG. C=O stretching was also observed at PLGA and LGseseTAPEG copolymer while CH_2_ stretching was clearly obtained at LGseseTAPEG copolymer. These results also showed the successful synthesis of LGseseTAPEG copolymer. For ^1^H-NMR analysis, PLGA, PEG, TA-PEG conjugates, PLGA-selenocystamine conjugates and LGseseTAPEG copolymer were dissolved in DMSO-d_6_. Selenocystamine was dissolved in D_2_O and mixed with DMSO-d_6_ (D_2_O/DMSO-d_6_ = 1/1, *v*/*v*) (data not shown).

### 3.2. Preparation and Characterization of CIP-Incorporated Nanoparticles

CIP-incorporated nanoparticles using LGseseTAPEG copolymer were prepared using the W/O/W/W double emulsion method. CIP contents were measured UV spectrophotometry as summarized as Table 1, where the experimental CIP contents in the nanoparticles were lower than the theoretical value, indicating that some of drug was lost during the drug-loaded nanoparticles fabrication process because CIP is a water-soluble drug. Furthermore, the loading efficiency was also decreased when the drug weight was increased, as shown in Table 1. The particle sizes of empty nanoparticles were 153 nm. However, the size of the nanoparticles became bigger when CIP was incorporated. The higher the CIP contents in the nanoparticles the bigger the particle sizes were.

**Figure 3:**
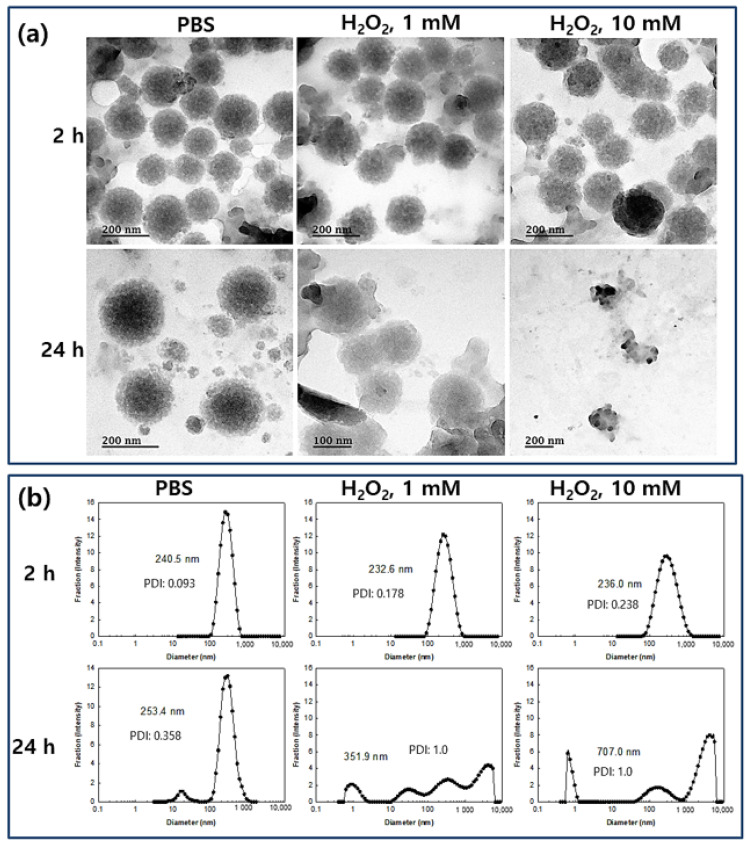
(**a**) TEM images and (**b**) typical particle size distribution of CIP-incorporated LGseseTAPEG nanoparticles. H_2_O_2_ was added to the nanoparticle aqueous solution (1 mg/mL (as a polymer weight) in 10 mL PBS) and then incubated them at 37 °C. For TEM observation and particle size analysis, CIP-incorporated nanoparticles (drug content: 10.1% (*w*/*w*)) were used. PDI: polydispersity index.

Kurutas et al. reported that the activity of markers for oxidative stress such as catalase and superoxide dismutase (SOD) were decreased while lipid peroxidation levels were increased [6]. They argued that extracellular liberation of ROS by phagocytic cells can be considered as one of the major factors to evaluate the severity of symptomatic infections and tissue damage in UTIs. To investigate the ROS-sensitivity of the nanoparticles, H_2_O_2_ was added to an aqueous nanoparticle solution and then incubated at 37 °C. After that, changes of morphology and particle size distribution were studied, as shown in Figure 3. As seen in Figure 3a, the morphology of nanoparticles was significantly changed and they disintegrated due to the addition of H_2_O_2_ to the nanoparticle aqueous solution although these changes of morphology were not significant at 2 h incubation. However, the nanoparticles were significantly disintegrated and distorted by addition of H_2_O_2_ and incubation for 24 h at 37 °C while nanoparticles in the control treatment (PBS at 24 h) maintained their spherical morphology as shown in Figure 3a. Furthermore, the size distribution of nanoparticles was also changed by the addition of H_2_O_2_, i.e., nanoparticles maintained a monomodal distribution pattern until 2 h of incubation even though their sizes were increased. After 24 h of incubation, the size-distribution of the nanoparticles became multi-modal patterns and the distribution pattern of the nanoparticles was distorted by the addition of H_2_O_2_ even though nanoparticles in PBS also showed a bimodal distribution pattern as seen in Figure 3b. These results indicate that LGseseTAPEG nanoparticles effectively respond to the oxidative stress and then their physical properties can be changed according to the intensity and duration of the oxidative stress. Kim et al., also reported that polymeric conjugates having diselenide linkages underwent ROS-sensitive changes in morphology, particle size and drug release behavior [27]. Like their nanoparticles, our nanoparticles also display ROS-sensitive changes of morphology, particle size distribution and drug release behavior.

**Figure 4:**
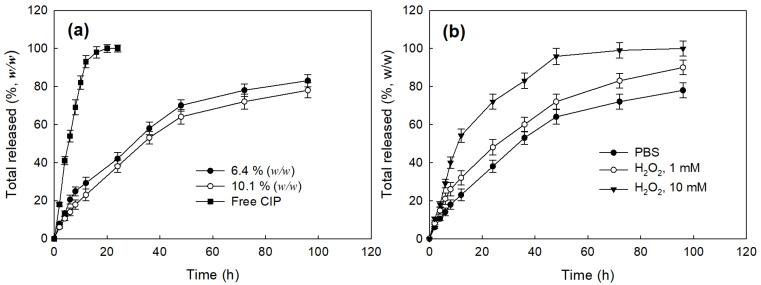
Drug release behavior of CIP-incorporated LGseseTAPEG nanoparticles. (**a**) The effect of drug contents and (**b**) the addition of H_2_O_2_ in the drug release media. To test the effect of H_2_O_2_, CIP-incorporated nanoparticles (10.1% (*w*/*w*)) was reconstituted into PBS in the presence or absence of H_2_O_2_. To assess the effect of H_2_O_2_ addition, CIP-incorporated nanoparticles (drug content: 10.1% (*w*/*w*)) were used.

Figure 4 shows the drug release profiles of the LGseseTAPEG nanoparticles. As shown in Figure 4a, CIP release from nanoparticles continued more than 4 days while free CIP was rapidly liberated and almost of drug was liberated after 24 h. When the drug content was increased, the drug release from the nanoparticles slightly decreased even though the differences of release rate was not significant (Figure 4a). In the case of free CIP, drug concentration in the media was significantly higher for 12 h and then drug concentration approached zero 24 h later while CIP-incorporated nanoparticles maintained detectable drug concentration until 96 h (Figure S1). The presence of H_2_O_2_ induced a faster CIP release from the nanoparticles and, furthermore, higher concentrations of H_2_O_2_ resulted higher drug release rates as shown in Figure 4b. Nanoparticles fabricated with LE copolymer, which does not have diselenide linkages, did not significantly respond to addition of H_2_O_2_ (Figure S2). At 10 mM H_2_O_2_, over 90% (*w*/*w*) of the drug was released at 48 h, indicating that LGseseTAPEG nanoparticles have an oxidative stress-sensitive drug release behavior. Figure 3 and Figure 4 indicate that LGseseTAPEG nanoparticles have ROS-sensitivity and potential for ROS-mediated drug delivery.

Jang et al., reported that nanofiber mats fabricated from ROS-sensitive polymers such as poly(L-lactide) (PLA)-PEG copolymer having diselenide linkages have sensitivity against H_2_O_2_ and then released piperlongumine in a ROS-sensitive manner [31]. They argued that drug release must be also accelerated by piperlongumine since piperlongumine is a ROS-producing agent. They showed that the ROS-sensitive release of piperlongumine effectively inhibited viability of cancer cells in vitro and in vivo. Fan et al., also reported that polymeric micelles composed of PLGA-PEG copolymer having diselenide linkages release berberine in a ROS-specific manner while berberine release in PBS was minimized [32]. Furthermore, it was reported that dexamethasone release from ROS-sensitive nanoparticles was accelerated in the presence of ROS due to the cleavage of diselenide linkages and this effectively inhibited the proliferation of activated macrophages [33].

### 3.3. Antibacterial Activity of CIP-Incorporated Nanoparticles

**Figure 5:**
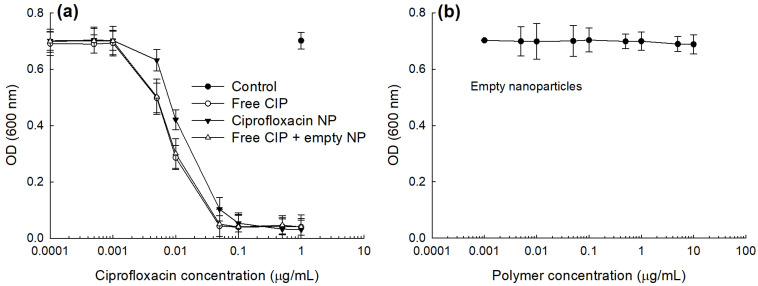
Antibacterial activity of CIP-incorporated nanoparticles. (**a**) Free CIP, CIP-incorporated nanoparticle (CIP-incorporated NP) or free CIP+empty nanoparticles (Free CIP + empty NP). For control, PBS was used to treat bacteria. (**b**) Empty nanoparticles. PBS (pH 7.4, 0.01 M) was used for control treatment. To assess the antibacterial activity of CIP-incorporated nanoparticles, CIP-incorporated nanoparticles (drug content: 10.1% (*w*/*w*)) were used.

To study the antibacterial activity of CIP and CIP-incorporated nanoparticles, *E. coli*, a typical uropathogen, was incubated with free CIP and CIP-incorporated nanoparticles as shown in Figure 5. As seen in Figure 5a, CIP and CIP-incorporated nanoparticles dose-dependently inhibited the growth of bacteria at higher than 0.001 μg CIP/mL while empty nanoparticles have practically no inhibitory effect until 10 μg polymer/mL against the growth of *E. coli* as shown in Figure 5b. Free CIP plus empty nanoparticles showed practically similar antibacterial activity compared to free CIP. CIP-incorporated nanoparticles showed lower antibacterial activity compared to free CIP or free CIP + empty nanoparticles. These results might be due to that CIP-incorporated nanoparticles have sustained release behavior and then the liberated CIP can directly affect the viability of bacteria. As shown in Table 2, IC_50_ values of CIP, CIP-incorporated nanoparticles and CIP + empty nanoparticles were 0.0084, 0.0188 and 0.0087 μg CIP/mL while the IC_50_ value of empty nanoparticles was higher than 10 μg polymer/mL.

In fact, in vitro studies cannot replace in vivo animal studies because all of the treated drug or nanoparticles is exposed to bacteria and participate in the inhibitory procedure of the bacteria. Systemic or local administration of CIP must resulted in rapid clearance of the drug from the body and then a minimal amount of drug remains in the body for inhibition of bacteria. However, nanoparticles have extended blood circulation properties and sustained drug release behavior. Therefore, these properties enable antibiotics to inhibit bacteria for a longer time [34].

**Figure 6:**
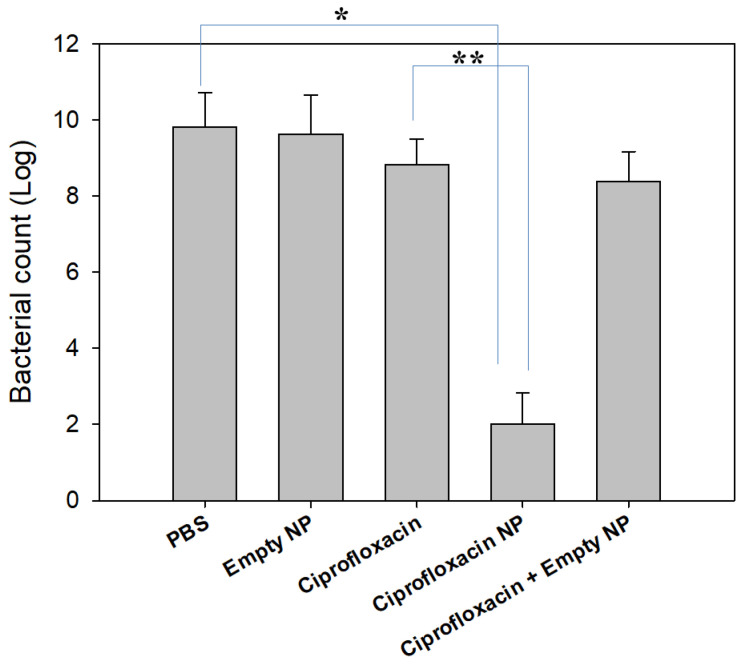
Antibacterial activity of CIP-incorporated nanoparticles. CIP (0.1 mg CIP/0.5 mL PBS) and nanoparticles in PBS solution (0.1 mg as a CIP concentration in 0.5 mL PBS) was introduced into dialysis membrane. Same quantity of empty nanoparticles or empty nanoparticles with free CIP in dialysis membrane were used to compare. For control treatment, 0.5 mL PBS in dialysis membrane was used. To assess the antibacterial activity of CIP-incorporated nanoparticles, CIP-incorporated nanoparticles (drug content: 10.1% (*w*/*w*)) were used. *, *p* < 0.01; **, *p* < 0.01.

From these points of view, we designed an antibacterial activity test with consideration of the drug release behavior of nanoparticles. Jeong et al., has reported that bacteria introduced into a dialysis membrane could be adapted to evaluate in vivo antibacterial activity [34]. Their method aims to mimic urinary tract infections in vivo. IN this study, we introduced CIP-incorporated nanoparticles into a dialysis membrane to mimic an in vivo antibacterial experiment and this system was placed into a bacterial culture. Free CIP dissolved in PBS and free CIP + empty nanoparticles were also introduced into the dialysis membrane and then used to compare the results, as shown in Figure 6, where CIP-incorporated nanoparticles showed significantly lower count of viable bacteria while empty nanoparticles did not affect the viability of bacteria. Free CIP and Free CIP + empty nanoparticles showed only a small decrease in bacteria viability compared to control. These results indicated that the CIP liberated from nanoparticles can affect the growth of bacteria continuously because nanoparticles have sustained release properties and remain in the dialysis membrane, and liberated CIP continuously affected the bacteria until the end of the culture period. On the other hand, CIP was immediately released from the dialysis membrane and this system was not able to maintain its antibacterial capacity for the whole period of bacteria culture because half of the media was exchanged with fresh media and then CIP was exhausted after a certain time. This system can reflect the sustained release behavior of nanoparticulate drug delivery system. Nanoparticulate-based drug delivery systems are suitable to inhibit biofilm formation [35,36,37,38]. It was reported that core-shell nanospheres-immobilized onto urinary catheters effectively inhibited *E. coli*-associated biofilm formation by up to 80% compared to control [37]. Furthermore, other scientists have developed a multilayer-coating of bacterial-responsive nanospheres onto urinary catheters that revealed superior inhibitory effect against biofilm formation [38]. Our investigation also showed that nanoparticles have superior inhibitory effect against the viability of bacteria and the ROS-sensitive drug release behavior of nanoparticles can be considered as a superior candidate for UTI treatment.

### 3.4. Intrinsic Cytotoxicity of LGseseTAPEG Nanoparticles against L929 Mouse Fibroblast Cells and CCD986sk Human Skin Fibroblast Cells

**Figure 7:**
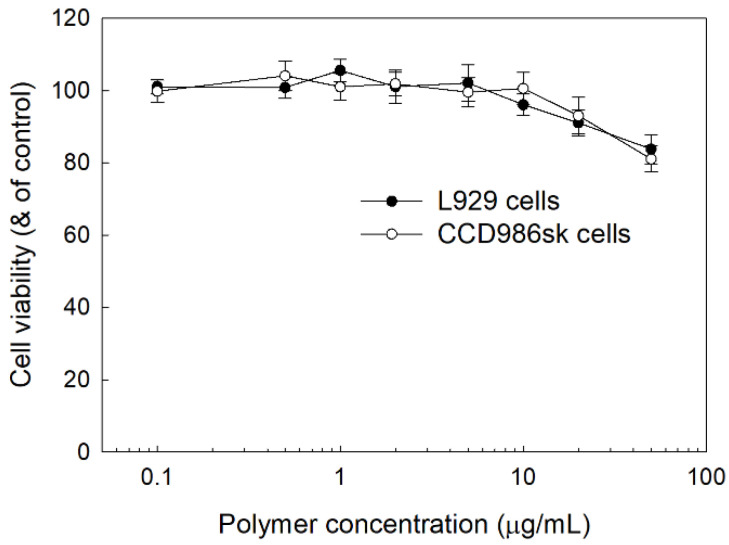
Cell cytotoxicity of LGseseTAPEG nanoparticles against L929 mouse fibroblast cells and CCD986sk human skin fibroblast cells. 2 × 10^4^ cells in 96 wells were exposed to nanoparticles of LGseseTAPEG copolymer for 24 h. Cell viability was evaluated with an MTT assay.

To study the intrinsic cytotoxicity of nanoparticles of LGseseTAPEG copolymer, L929 mouse fibroblast cells and CCD986sk human skin fibroblast cells were used. As shown in Figure 7, the viability of L929 cells and CCD986sk cells remained higher than 90% until 10 μg polymer/mL. At 50 μg polymer/mL, viability was still higher than 80%, both for L929 cells and CCD986sk cells. These results indicated that nanoparticles of LGseseTAPEG copolymer have no acute toxicity against normal fibroblast cells and they might be compatible with normal cells.

## 4. Conclusions

LGseseTAPEG copolymer containing diselenide linkages was synthesized for ROS-sensitive drug delivery. Synthesis of LGseseTAPEG copolymer was confirmed using ^1^H- NMR spectroscopy and FT-IR spectroscopy. CIP-incorporated nanoparticles of LGseseTAPEG copolymer were fabricated by a W/O/W/W emulsion method. CIP contents in the nanoparticles were 6.4% (*w*/*w*) and 10.1% (*w*/*w*). CIP-incorporated nanoparticles responded to H_2_O_2_ and their morphologies disintegrated during 24 h incubation in the presence of H_2_O_2_. Furthermore, the particle size distribution changed from a monomodal distribution pattern to a multimodal distribution pattern by addition of H_2_O_2_ to aqueous solutions of nanoparticles. Drug release from the nanoparticles also revealed ROS-sensitive behavior, i.e., the drug release rate was significantly increased in the presence of H_2_O_2_. These results indicated that physicochemical properties of nanoparticles were changed by addition of H_2_O_2_ and these nanoparticles have ROS-sensitivity. In an antibacterial study using *E. coli*, free CIP and free CIP plus empty nanoparticles showed dose-dependent inhibitory effects against the growth of bacteria while CIP-incorporated nanoparticles have less antibacterial activity compared to free CIP. These results were due the fact that CIP-incorporated nanoparticles have sustained release properties. Free CIP and CIP-incorporated nanoparticles were introduced into a dialysis membrane to mimic the in vivo situation. In this experiment, CIP-incorporated nanoparticles showed superior antibacterial activity while free CIP did not significantly inhibit the growth of bacteria. In a cell viability assay, nanoparticles of LGseseTAPEG copolymer displayed no acute cytotoxicity against L929 mouse fibroblast cells or CCD986sk human skin fibroblast cells. We suggest LGseseTAPEG nanoparticles are a promising candidate for CIP delivery.

## Figures and Tables

**Table 1:** Drug contents and particle size of ciprofloxacin-incorporated nanoparticles.

Polymer/Ciprofloxacin (mg/mg)	Drug Contents (%, *w*/*w*)		Loading Efficiency (%, *w*/*w*) ^a^	Particle Size (nm) ^b^	
	Theoretical ^a^	Experimental ^a^		Average Diameter ± S.D.	PDI ^c^
100/0	-	-	-	153 ± 7.94	0.065
100/10	9.1	6.4	68.4	238 ± 15.6	0.125
100/20	16.7	10.1	56.1	318.4 ± 22.5	0.158

^a^ Drug contents (%, *w*/*w*) were calculated as follows: Theoretical (%, *w*/*w*) = [Feeding weight of drug/(feeding weight of drug + feeding weight of polymer)] × 100. Experimental = (Practical drug weight in the nanoparticles/total weight of nanoparticles) × 100. Loading efficiency = (Feeding weight of drug/ Practical drug weight in the nanoparticles) × 100. ^b^ Particle sizes of nanoparticles were measured at least three times. ^c^ Polydispersity index.

**Table 2:** IC_50_ values of ciprofloxacin-incorporated nanoparticles against bacteria.

Drug or NP Treatment ^a^	IC_50_ (μg CIP/mL)
Free CIP	0.008
CIP-incorporated NP	0.019
Free CIP + empty NP	0.009
Empty NP ^b^	>10

^a^ NP, nanoparticles. ^b^ Empty NP was (μg polymer/mL).

## Data Availability

Data sharing not applicable.

## Supplementary Materials

The following are available online at https://www.mdpi.com/article/10.3390/ma14154125/s1, Figure S1: Time course of released CIP concentration in the media. CIP-incorporated nanoparticles of LGseseTAPEG copolymer were used to analyze drug release study. Figure S2: CIP release from LE block copolymer nanoparticles. The effect of the addition of H_2_O_2_ in the drug release media. To test the effect of H_2_O_2_, CIP-incorporated nanoparticles were reconstituted into PBS in the presence or absence of H_2_O_2_.
