# Supplementary material for: Ciprofloxacin-Releasing ROS-Sensitive Nanoparticles Composed of Poly(Ethylene Glycol)/Poly(D,L-lactide-co-glycolide) for Antibacterial Treatment
Source: Materials (Basel). 2021 Jul 24;14(15):4125. doi: 10.3390/ma14154125 (PMC8348395; doi:10.3390/ma14154125)
Supplement: Supplementary file 1 [file materials-14-04125-s001.zip › materials-1266877-SI.pdf]

materials

MDPI

Supporting Information

Ciprofloxacin-Releasing ROS-Sensitive Nanoparticles Composed of Poly(Ethylene Glycol)/Poly(D,L-lactide-co-glycolide) for Antibacterial Treatment

Jaeik Song [1], Min-Suk Kook [2], Byung-Hoon Kim [3], Young-IL Jeong [3] and Kyung-Jin Oh [1,*]

[1] Department of Urology, Chonnam National University Medical School, Chonnam National University Hospital, Gwangju 61469, Korea; puzzlelist@naver.com
[2] Department of Maxillofacial Surgery, School of Dentistry, Chonnam National University, Gwangju 61186, Korea; omskook@chonnam.ac.kr
[3] Department of Dental Materials, School of Dentistry, Chosun University, Gwangju 61452, Korea; kim5055@chosun.ac.kr (B.-H.K.); nanomed@naver.com (Y.-I.J.)
* Correspondence: okj1225@jnu.ac.kr; Tel.: +82 62 220 6704

Citation: Song, J.; Kook, M.-S.; Kim, B.-H.; Jeong, Y.-I.; Oh, K.-J. Ciprofloxacin-Releasing ROS-Sensitive Nanoparticles Composed of Poly(Ethylene Glycol)/Poly(D,L-lactide-co-glycolide) for Antibacterial Treatment. *Materials* **2021**, *14*, 4125. https://doi.org/10.3390/ma14154125

Academic Editor: Daniela Berger

Received: 3 June 2021
Accepted: 20 July 2021
Published: 24 July 2021

Publisher's Note: MDPI stays neutral with regard to jurisdictional claims in published maps and institutional affiliations.

Copyright: © 2021 by the authors. Licensee MDPI, Basel, Switzerland. This article is an open access article distributed under the terms and conditions of the Creative Commons Attribution (CC BY) license (http://creativecommons.org/licenses/by/4.0/).

1. Experimental

1.1. Materials

PLGA/PEG (LE) block copolymer (PEG M_n: 5,000 g/mol; PLGA M_n: 20,000 g/mol; lactide:glycolide raio of PLGA, 50:50) was purchased from Sigma-Aldrich Co. (St. Louis, MO, USA).

1.2. Preparation of CIP-incorporated LGseseTAPEG nanoparticles

CIP-incorporated nanoparticles were fabricated as follows: LGseseTAPEG copolymer (100 mg) was dissolved in 10 ml of DCM and CIP (10 mg or 20 mg) was dissolved in 1 ml deionized water. These solutions were mixed and vigorously sonicated with ultrasonicator (40 W, 1 min, Vibracell VCX 400, Sonics & Materials Inc., Newtown, CT, USA) to make a water in oil (W/O) emulsion. This solution was poured into 15 mL of aqueous PVA solution (1%, *w/v*) and then vigorously homogenized (HG-15A, Daihan Scientific, Seoul, Korea) at 15,000 rpm for 1 min. The mixture was sonicated again with ultrasonicator to make a water-in-oil-in water (W/O/W). This emulsion solution was poured into 50 mL of PVA solution (0.5%, *w/v*) and then stirred with an overhead stirrer at 1000 rpm (Direct Driven Digital Stirrer, SS-11D, Young HANA Tech. Co., Seoul, Korea) for 90 min. Following this, this solution was centrifuged to harvest CIP-incorporated nanoparticles using a vacuum high speed centrifuge at 15,000 rpm (Supra 30K, Hanil Science Industrial Co. Ltd., Seoul, Korea). To remove surfactant or un-incorporated drugs, harvested nanoparticles were susoended in deionized water once more and then harvested again using a vacuum high-speed centrifuge at 15,000 rpm. These were finally lyophilized for more than 2 days.

To measure the CIP contents in the nanoparticles, 10 mg of lyophilized solids were dissolved in 5 mL DCM and, after that, 2 mL of deionized water was added. This mixture was magnetically stirred for more than 5 h and then 0.5 mL of water phase was used to measure the CIP concentration at 277 nm using a UV-VIS spectrophotometer (UV-VIS spectrophotometer 1601, Shimadzu Co. Tokyo, Japan) at 340 nm. Drug contents were calculated as follows:

Drug contents = (drug weight/nanoparticle weight) × 100. Loading efficiency = (Initial feeding amount of drug weight/remained drug weight in the nanoparticles) × 100. (1)

1.3. Preparation of CIP-incorporated LE block copolymer nanoparticles

CIP-incorporated LE block copolymer nanoparticles were fabricated as follows: LE block copolymer (100 mg) was dissolved in 10 mL of DCM and CIP (20 mg) was dissolved in 1 mL deionized water. These solutions were mixed and vigorously sonicated with an ultrasonicator (40 W, 1 min, Vibracell VCX 400, Sonics & Materials Inc.) to make a water in oil (W/O) emulsion. This solution was poured into 15 mL of aqueous PVA solution (1%, *w*/*v*) and then vigorously homogenized (HG-15A, Daihan Scientific, Seoul, Korea) at 15,000 rpm for 1 min. This mixture was sonicated again with the ultrasonicator to make a water-in-oil-in water (W/O/W). This emulsion solution was poured into 50 mL PVA solution (0.5%, *w*/*v*) and then stirred with an overhead stirrer at 1000 rpm (Direct Driven Digital Stirrer, SS-11D, Young HANA Tech. Co., Seoul, Korea) for 90 min. Following this, this solution was centrifuged to harvest CIP-incorporated nanoparticles using a vacuum high speed centrifuge at 15,000 rpm (Supra 30K, Hanil Science Industrial Co. Ltd., Seoul, Korea). To remove surfactant or un-incorporated drugs, the harvested nanoparticles were susoended in deionized water once more and then harvested again using the vacuum high-speed centrifuge at 15,000 rpm. Th final product was lyophilized for more than 2 days.

To measure CIP contents in the nanoparticles, 10 mg of lyophilized solids were dissolved in 5 mL DCM and, after that, 2 mL of deionized water was added. This mixture was magnetically stirred for more than 5 h and then 0.5 mL of water phase was removed and used to measure the CIP concentration at 277 nm using a UV-VIS spectrophotometer (UV-VIS spectrophotometer 1601, Shimadzu Co. Tokyo, Japan) at 340 nm. Drug contents were calculated as follows:

Drug contents = (drug weight/nanoparticle weight) × 100. (2)

From the measurements of drug contents, the CIP content in the LE block copolymer nanoparticles was approximately 9.3% (*w*/*w*).

1.4. Drug release from nanoparticles

CIP release from nanoparticles was determined as follows: nanoparticles (10 mg) were distributed in 3 mL of phosphate buffered saline (PBS, pH 7.4, 0.01 M) and then put into a dialysis membrane (MWCO, 8,000 g/mol). Following this, this was placed in a 50 ml Falcon tube with 47 mL of PBS and then stirred at 100 rpm (37 °C). Whole media were exchanged at predetermined time intervals to prevent saturation of the drug and then the released drug was measured with a UV-spectrophotometer (UV-1601, Shimadzu Co. Ltd.) at 277 nm. The following expression ws used for the calculations:

CIP concentration vs time (μg/mL/h) = (Drug concentration measured by UV spectrophotometer/time intervals for measurement of drug concentration). (3)

2. Results

Figure S1 shows the CIP concentration versus time. In Figure S1, a burst release of CIP from nanoparticles was observed until 24 h and then release was continuous until 96 h. As shown in Figure S1a, CIP concentration vs time was highest for free CIP and then 10.1% (*w*/*w*) drug contents was slightly higher than that of 6.4% (*w*/*w*). When H_2O_2 was added, the highest CIP concentration in the release media was observed at 10 mM H_2O_2 than that of PBS or 1.0 mM H_2O_2.

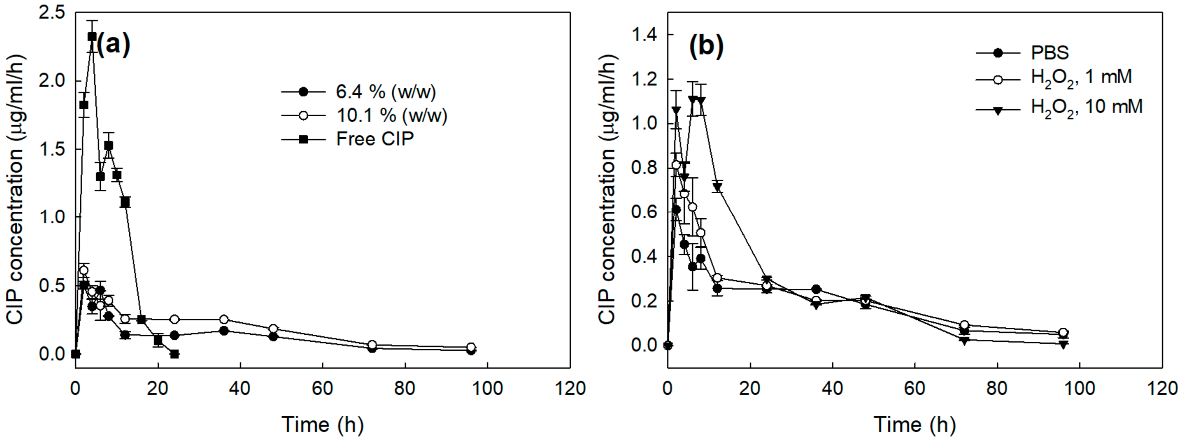

Figure S1. Time course of released CIP concentration in the media. CIP-incorporated nanoparticles of LGseseTAPEG copolymer were used to analyze drug release study.

Figure S2 shows the CIP release from LE block copolymer nanoparticles. As shown in Figure S2, the CIP release rate was not significantly changed by the addition of H_2O_2 while CIP release from LGseseTAPEG copolymer nanoparticles was significantly affected by the addition of H_2O_2 (Figure 4b). These results indicated that LGseseTAPEG copolymer nanoparticles have ROS-sensitive drug release potential.

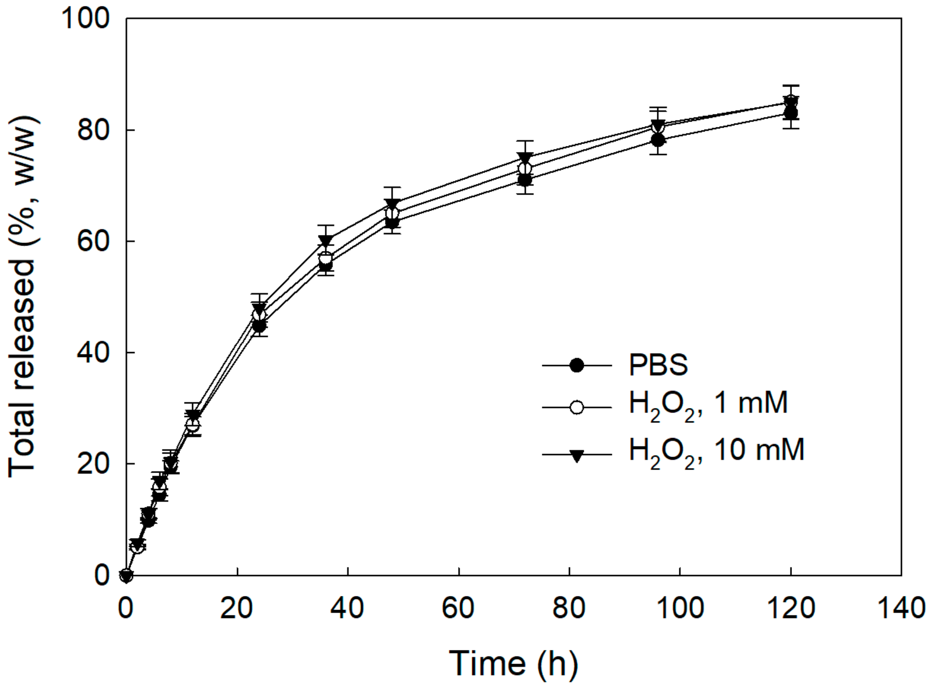

Figure S2. CIP release from LE block copolymer nanoparticles. The effect of the addition of H_2O_2 in the drug release media. To test the effect of H_2O_2, CIP-incorporated nanoparticles were reconstituted into PBS in the presence or absence of H_2O_2.
